# A Few Remarks on Foreign Bodies in the External Auditory Meatus

**Published:** 1883-07

**Authors:** F. C. Hotz

**Affiliations:** Chicago


### ARTICLE III.

### A FEW REMARKS ON FOREIGN BODIES IN THE EXTERNAL AUDITORY MEATUS. By F. C. HOTZ, M.D., Chicago.

Although, as a general rule, it is advisable to remove foreign bodies in the external auditory meatus at an early day. we need not be unduly hasty about it; for the most foreign bodies that accidently get into the meatus, if left alone, may remain there for an indefinite period without doing any harm. So, for instance, Politzer (in his Text Book) mentions the case of an old man from whose ear he took a plug of inspissated cerumen, embedded in which he found the piece of a slate pencil which the patient had put in his ear fifty years before. Similar observations are recorded in literature in sufficiently large number to attest the harmlessness of foreign bodies in the external auditory meatus.

But when, by some other cause, the ear becomes inflamed, the presence of a foreign body is apt to intensify the inflammation. This, for instance, was shown in the case of a boy, aged eight years, who was brought to me by his mother, in April, with the following history: Three years before, after measles, he had inflammation of the right ear with a slight discharge, which lasted three or four days; afternoon he complained of earache very often when he had a cold in his head, but there was no discharge from the ear. Four days ago he woke up in the night with violent pain in the right ear; during the day it subsided, but became very severe again the next night. The following day the ear began to discharge a muco-purulent fluid tinged with blood, but the earache did not subside. Yesterday his mother noticed a fleshy growth in the ear, which by this morning had grown so large as to fill the entire orifice of the meatus.

I found the entrance of the external meatus plugged by a smooth, pretty consistent polypus, as large as a pea, and attached to the floor of the cartilaginous portion of the meatus. I removed it at once with the wire snare and touched the place it sprang from with chromic acid. The deeper portion of the auditory canal was filled with what seemed to be inspissated pus and earwax; but as the removal of the polypus had given the boy a great deal of pain, so that he began to be uneasy and nervous, I considered it prudent to abstain from further manipulations for the present, and plugged the meatus with absorbent cotton soaked with a saturated solution of boracic acid. When the boy came back next day, the cotton plug was quite dry and just ready to drop out of the auditory canal, and upon removing it, I saw in the meatus, near the external orifice, a roundish, black body, which could easily be seized with the forceps, and proved to be a large seed of a watermelon. This seed must have advanced toward the entrance of the meatus during the night, for on the previous day it lay in the deepest portion of that canal, where its dark color, mixed with the pus and granulations around it, made the whole mass look like inspissated wax and pus. After the meatus was cleansed, its walls presented the rose red surface characteristic of otitis diffusa. The membr. tymp. also was quite red, and anterior and parallel to the handle of the malleus there was a small, oblong perforation, with smooth and indurated edges, through which a muco-purulent secretion oozed out. A few insufflations of impalpable boracic acid arrested the secretion and restored the natural state of the external meatus, but the perforation of the membr. tympani did not close.

This perforation presented all the signs of an old lesion, and was evidently the product of the otitis media catarrhalis the boy had three years ago. How and where the melon seed got in his ear, the boy could or would not tell; but we may presume that it has been in the deeper part of the meatus probably since last sum mer without causing any inconvenience. But when recently the otitis media was started up again by a cold (of which the nose and throat showed ample evidence), the presence of the seed favored the propagation of the inflammation from the tympanic cavity to the external auditory meatus, and undoubtedly was the chief cause of the rapid growth of polypoid granulations upon the raw integument of the auditory canal. But for this otitis media, the seed might have remained in the ear unnoticed for a long time.

It is very well to remember this fact of the harmlessness of the most foreign bodies in a healthy ear, particularly when the patient is a child (and the majority of these cases are children), and an over-anxious mother is urging the doctor to remove the foreign body at all hazards. If, under such circumstances, the physician does not proceed with calm deliberation, weighing carefully the chances of succeeding with removing a foreign body from the ear of a child intensely scared by the excited talk and actions of his mother; if he makes improper, rash attempts at the removal of the foreign body, he is apt to do greater harm to the ear, and to inflict more pain upon the child than the foreign body ever is likely to do. The external auditory meatus cannot be syringed properly if the head is moved just at the moment when the water is thrown into the meatus, and by no means is it safe to introduce an instrument (for instance, a sharp hook, which is a very ~~unsafe~~ instrument for removing beans, peas, etc.,) into the depth of the meatus while the child may jerk his head.

If, therefore, a child is brought to a physician for the removal of a foreign body from the ear, and he does not succeed by a few attempts with the syringe, because the child struggles, he should desist from further endeavors, unless he is prepared to put the child under the influence of an anæsthetic. It always has proven, and always will prove a sad mistake to persist by brute force in trying to remove the foreign body when the child is unmanageable from fear or any other cause. These improper attempts will only injure the ear, and drive the foreign body deeper into the ext. auditory meatus, way through the membr. tympani into the middle ear.

For instance, in August, Viola K., a delicate girl, aged three years, was brought to my office by her parents, who were in a state of the greatest anxiety. One week before, the child had put a dry grain of corn in her right ear. A physician was summoned immediately. After a few unsuccessful attempts with forceps, during which the ear began to bleed, he chloroformed the child, syringed the ear, and assured the parents the corn had come out with the water. But the mother asserted positively she had seen the yellow body in the ear on the day before they came to me Whether she was right or the physician, I could not determine at once, for the ear was so tender that a proper examination without chloroform was out of the question ; and the integument of the ext. meatus was so swollen, that even under narcosis, its deeper portion could not be inspected, and as I regarded this violent otitis as the result of improper surgical interference rather than as being produced by the foreign body, I desisted from any further examination for the present, and prescribed a solution of boracic acid, to be dropped into the ear three or four times per day.

As I expected, the inflammation readily subsided under this treatment, but the child did not come back until October 12. The meatus was then in a normal condition, and I could easily see the yellow dry corn in the depth of the meatus upon the membr. tympani. The child, however, was so scared by past experience, that I put it under the influence of chloroform. A small, sharp hook passed behind the corn brought it out with the greatest facility.

In this connection, one other instance may be briefly related, in which the ear was more seriously injured by imprudent endeavors to remove the foreign body: Jim B., six years old, was brought to my office September 6, in the afternoon. In the morning he had put one of his incisor teeth that had just fallen out into his right ear. His mother took him at once to a physician, who, as she put it, " worked at the ear until it bled awfully, but he did not get the tooth." After the blood clots were removed from the meatus by means of absorbent cotton. I could see the white tooth on the membr. tymp., but the ear was very sensitive, so much so that the syringing caused a great deal of pain. I therefore put the boy under the influence of chloroform, when I had not the least difficulty in removing the tooth. I found then that the membr. tymp. was perforated at the point where the tooth had laid against it. This perforation was caused by the sharp point of the tooth where it was forced down into the meatus by the ill-advised manipulations of the physician. No permanent damage, however, resulted from it, for the ear did not become inflamed, and the perforation healed in the course of one week.

Be sure that the foreign body is in the ear before you proceed to remove it. This proposition is so self-evident that any further comment seems superfluous. And yet, to use Dr Roosa's words,* "ears have been syringed—much worse, have been treated with instruments; membranæ tympani have been broken; ossicles have been dragged out, and meningitis and death have been caused by neglect of this simple rule." And why is this simple rule neglected? Because physicians often take it for granted that the foreign body must be in the ear of the patient, as his parents and friends say so. But we should not take their word for it, but should ascertain its presence by a proper examination; we should not rely upon what we are told, but upon what we can see. Now, this must not be understood as if I meant to say that patients habitually conceal the truth, or purposely tell a lie. I believe that patients, with few exceptions, give us their story as truthfully as they know it, and we seldom have an occasion to doubt their veracity. But in the matter of foreign bodies, we must not accept the patient's word, or the statement of anybody else, as sufficient evidence, because they themselves may be mistaken, or the foreign body which they have felt or seen in the ear or eye, may have come out by the time they consult a physician. Just as we sometimes find a foreign body in the eye or ear, while the patient was not aware of its presence, so we meet patients who are positively convinced that there is a foreign body in the eye or ear, when the proper examination shows there is none there. How firm this belief may sometimes be, the following case may show:

Last year, in March, a young lady came to me to have a pin removed from her right ear. Her mother, who came with her, was so excited that she could scarcely compose herself enough to tell me what had happened. At last, however, I learned that her daughter, the previous day, used a pin to relieve a disagreeable

---

* *N. Y. Med. Record*, Dec. 10, 1881.

itching in her right ear, and while she was scratching the meatus with the head of the pin, it slipped from her fingers, and as she could not find it anywhere, she concluded it must be in the ear. They went at once to a physician, who, upon the opinion of the women, and without examination, operated upon the ear with forceps to extract the pin. After several unsuccessful attempts, he gave up, saying he could feel the pin, but his forceps could not grasp it tightly enough. He had evidently mistaken a projection of the osseous meatus for the pin; for when I examined the ear, I found no pin in it, but saw several abrasions of the osseous meatus. The mother was quite distracted when I did not produce the pin; since that physician had told her he could feel the pin, she was thoroughly convinced that it was there, and when I declared I could not see a pin in the ear, and that I thought it had dropped out, she would not accept this explanation, but concluded that it had gone further into the head of her poor daughter; she saw it already traveling through the brain, and pictured to herself the most terrible consequences. Fortunately, however, the young lady did not get sick, and after a few days her mother had overcome her grief and anxiety.

Such and similar occurrences may be my apology for closing this paper with the following remarks: When we consider how little trouble it is to make a proper examination of the ear and meatus, and when we consider how much suffering a physician can relieve by intelligent treatment, based upon a proper examination of the ext. aud. meatus—is it then not very strange that as yet comparatively so few physicians are in the habit of making a proper examination when they are consulted upon some affection of the ext. auditory meatus. Why is it? The proper examination of the meatus requires nothing more than a good illumination of this canal. Now, this does not require any special skill or training; it does not require any costly apparatus, and it does not require much time. A few dollars will buy the necessary instruments (reflector and funnel specula), and anybody with good eyesight can make the examination in a few minutes. There is, therefore, no more excuse for a physician to attend to a difficulty of the ear and meatus without the proper examination, than there is for him to diagnosticate and prescribe for pneumonia without auscultation and percussion of the chest. It is no greater trouble to carry a reflector than a stethoscope; but there is no doubt that the former can be used to much greater *direct* benefit of patients than the latter. It renders the interior of the meatus accessible to our view, so that any operation or medicinal application can be made as easily and precisely as if done upon the external integument. Without the aid of the reflector, the introduction of an instrument into the deeper portion of the meatus is about as prudent and safe as fast sailing in a dense fog; you are likely to strike a reef when you least expect it.

