# Errata

**Published:** 1883-08

**Authors:** 


ERRATA.

The following corrections should be made in the article by Dr. F. C. Hotz, of Chicago, published in our last issue, and entitled "A Few Remarks on Foreign Bodies in the External Auditory Apparatus." The author was unfortunately out of the city when the JOURNAL went to press:

Page 20, 7th line from below, read *afterwards* instead of afternoon.

Page 21, 16th line from below, read *raw* instead of rose.

Page 22, line 21, read *useful* for unsafe; and, on page 25, the words "ear and meatus" are used several times for *ext. audit meatus.*

